# Trauma-Informed Care in the Neonatal Intensive Care Unit: Through the Lens of the COVID-19 Pandemic

**DOI:** 10.7759/cureus.30307

**Published:** 2022-10-14

**Authors:** Shreyas Arya, Ankita Zutshi

**Affiliations:** 1 Department of Pediatrics, Wright State University Boonshoft School of Medicine, Dayton, USA; 2 Division of Neonatal-Perinatal Medicine, Dayton Children’s Hospital, Dayton, USA; 3 Department of Psychiatry and Behavioral Neuroscience, University of Cincinnati College of Medicine, Cincinnati, USA; 4 Division of Child and Adolescent Psychiatry, Cincinnati Children’s Hospital Medical Center, Cincinnati, USA

**Keywords:** covid-19 retro, pandemic, nicu, neonate, trauma-informed care

## Abstract

Trauma is rooted in an individual’s experience of an event that leads to physical or mental harm and can have a long-lasting, unfavorable effect on their well-being and functioning. Being aware of the effects of trauma, recognizing its signs, understanding how it informs individual responses, and actively trying to prevent re-traumatization are the tenets of trauma-informed care. Admission to the neonatal intensive care unit (NICU) is widely considered to be an extremely stressful time for parents and infants alike. With the emergence of the coronavirus disease 2019 (COVID-19) pandemic, there were significant changes in healthcare delivery. Widespread closures, restrictions due to infection control measures, the spread of misinformation, increased psychosocial hardships, and amplification of cultural, gender, and racial biases intensified NICU-related stressors. Adoption of the principles of trauma-informed care, as defined by the Substance Abuse Mental Health Services Administration, to the NICU can help buffer some of these stressors. We present a review of these principles viewed through the lens of the COVID-19 pandemic. The lessons learned will help inform practices and policies and allow us to navigate similar challenges more effectively in the future.

## Introduction and background

Trauma results from events and circumstances experienced by an individual which cause physical or mental harm and can have lasting negative effects on their functioning and well-being. The Substance Abuse Mental Health Services Administration (SAMHSA) conceptualized trauma using the 3Es: trauma is based on an individual’s “Experience” of an “Event” that leads to adverse “Effects” [1].

Delivering care through a trauma-informed lens means (a) being cognizant of the significant effect trauma has on patients, families, and healthcare professionals; (b) recognizing the signs of trauma and integrating these practices into hospital policies; (c) being perceptive of how trauma informs an individual’s responses to future traumatic situations; and (d) using this understanding to actively prevent re-traumatization [2].

In a landmark paper in 2012, Shonkoff et al. emphasized that experiencing trauma and toxic stress early in life can lead to epigenetic changes which affect future neurodevelopment and health [3]. Critically ill infants are subject to a myriad of traumatic stressors in the neonatal intensive care unit (NICU) [4,5]. These stressors have been shown to impact both short [6] and long-term outcomes [7,8]. During the coronavirus disease 2019 (COVID-19) pandemic, the protective element of intimate contact between newborns and their families was severely interrupted. In addition, the limited presence of support staff and widespread psychosocial hardships contributed to the collective trauma burden of society [9]. Healthcare workers also experienced a significant increase in mental health disorders, with those working in the intensive care settings being disproportionally affected [10]. This makes the adoption of the principles of trauma-informed care even more relevant in the NICU.

## Review

Key principles of trauma-informed care and their applications in the NICU

SAMHSA laid out six key principles to guide trauma-informed care across different clinical settings [1], which were adapted to the NICU by Sanders and Hall [11].

Safety for Patients, Caregivers, and Staff

Nurturing an environment that promotes a sense of safety, as perceived by the person being served. Applications of this principle include providing privacy to patients and caregivers (e.g., single-family room setups), being mutually respectful, and ensuring confidentiality.

Trustworthiness and Transparency

Building and maintaining trust and delivering care in a transparent manner. Applications of this principle include family-centered rounds, nursing assignments to ensure continuity of care, and frequent communication without the use of complicated medical terminology.

Peer Support and Mutual Self-Help

Peers are individuals who have experienced similar traumatic situations. Through shared understanding, support, and empowerment, they can positively influence treatment beyond the clinical setting. This can be done in person or remotely via the phone or internet and can be done individually or in a group. Applications of this principle include NICU parent support groups (e.g., the NICU parent network [12]) and rare diseases support groups (e.g., the Support Organization for Trisomy 18, 13, and Related Disorders, SOFT [13]).

Collaboration and Mutuality

Partnering with parents for care delivery and eliminating hierarchies in the healthcare setting. The goal is to recognize the therapeutic role each person plays in treatment. Applications of this principle include shared decision-making and collaboration between nurses, physicians, and the support staff.

Empowering Patients, Parents, and Staff in Having a Voice and a Choice

Providing an organizational framework that empowers patients, parents, and staff by recognizing each person’s experience and building upon their strengths. Supporting parents to cultivate self-advocacy skills and appreciating that staff are facilitators, rather than controllers, of treatment-related decision-making [14]. Applications of this principle include family-integrated care, providing psychosocial support to parents aimed at fostering resilience, and supporting staff scheduling and assignment requests.

Recognizing Cultural, Historical, Racial, and Gender-Based Differences

Actively recognizing biases that may be based on stereotypes and moving past them to provide equal care to all, regardless of age, religion, gender identity, sexual orientation, race, national origin, cultural differences, or disabilities. Applications of this principle include practicing cultural humility [15], being aware of generational trauma, and treating all patients and parents as equals regardless of their backgrounds.

Challenges to trauma-informed care in the NICU during the COVID-19 pandemic

Preterm infants account for approximately 10% of all births worldwide [16]. The majority of these infants require some duration of NICU stay. Among these infants, those born with congenital anomalies or extremely premature remain in the hospital for extended periods. NICU admissions are widely considered to be an extremely stressful time for parents and families. The emergence of the COVID-19 pandemic has led to significant changes in the delivery of healthcare. The impact of COVID-19-related restrictions on the care of infants and parent experience in the NICU has been extensively reported [9,17,18]. These studies found that changes to care during the pandemic amplified NICU-related stressors and eliminated a large number of protective factors, posing a direct challenge to the cornerstones of trauma-informed care.

Decreased Sense of Safety

Pregnancy is a stressful life event and the limited presence of partners or support persons during antenatal visits and childbirth contributed to mothers feeling isolated and distressed [19]. The majority of NICUs allowed only one parent at their infant’s bedside, while some units restricted all parents and caregivers, even if the child was in extremis [9,17]. While single-family room design helped preserve parental presence to some extent, these setups were not ubiquitous. Additionally, a disproportionate number of deaths during the COVID-19 pandemic were in the elderly population, reducing the safety net that grandparents provide in the care of infants and young children.

Lack of Trustworthiness and Transparency

Misinformation and widespread inequities created a sense of institutional distrust, the brunt of which was borne by healthcare professionals [20]. Universal masking and the use of personal protective equipment added additional barriers for physicians to make meaningful connections with parents and families. Participation of parents during family-centered NICU rounds was significantly decreased, limiting effective communication and overall feelings of trust [9].

Paucity of Peer Support

Due to widespread closures related to the COVID-19 pandemic, NICU parents had significantly reduced access to in-person peer support groups [21]. Mahoney et al. found that nearly half of the NICUs reported a reduction in the presence of social workers and ancillary support staff, who often play a vital role in identifying parents in need of support and connecting them with appropriate organizations and resources [9].

Absence of Collaboration and Mutuality

Prior to the COVID-19 pandemic, the majority of NICUs conducted family-centered multidisciplinary rounds in which parents, families, and a full team of staff participated in shared decision-making at the infant’s bedside. This model of care has been recommended by the American Academy of Pediatrics (AAP) [22], and the involvement of families in decision-making has been shown to reduce stress and improve outcomes [23]. However, due to restrictions on the presence of parents and nonessential staff, more than half of the NICUs in the United States reported interruptions in this model, thereby hindering opportunities for collaboration and mutuality [9].

Disruptions to the Framework of Empowerment

In an international survey, Kostenzer et al. found that a significant number of NICU parents reported feeling uninvolved in the care of their babies during the COVID-19 pandemic. Over half of the participants in their survey felt that they were not adequately informed and there was a lack of mental health support during this challenging time [17]. Moreover, these restrictions led to feelings of anger, sadness, and fear in parents [24] and increased the risk for postnatal depression and posttraumatic stress [25]. This caused major disruptions to the framework of parent empowerment.

Amplification of Cultural, Gender, and Racial Biases

The fear surrounding the COVID-19 outbreak amplified cultural and racial biases, both in the healthcare setting and the society as a whole. This ranged from seemingly innocuous acts of microaggressions to overt physical violence against people of Asian origin [26]. Widespread closures and government policy in response to the pandemic also had a disproportionately large effect on the health of people belonging to lower socioeconomic strata [27]. Even healthcare workers experienced an exacerbation in violence and fear-based discrimination [28].

Finally, studies reported that women faced more severe symptoms of mental health disorders during the COVID-19 pandemic [29]. At baseline, one in seven women reports suffering from anxiety and depression in the perinatal period, and this number is significantly higher in women with a medically high-risk pregnancy [30]. Lack of social support and decreased access to resources, during the pandemic, substantially exacerbated these risks.

## Conclusions

The word “pandemic” originates from the Greek word *pan* meaning “all” and *dēmos* meaning “people,” in that it has far-reaching effects on everyone. Therefore, we recommend a universal trauma-informed approach for all NICU parents, families, and staff. The emergence of the COVID-19 pandemic has presented unprecedented challenges to the delivery of trauma-informed care in the NICU. The development of enhanced online and telemedicine resources has mitigated some of these effects. However, restrictions due to strict infection control measures, while essential and inevitable, continue to remain a hindrance. The impact of these disruptions may become apparent in the years to come, as the world recovers from the pandemic. By understanding the importance of the concepts of trauma-informed care and incorporating them into policy, neonatal providers can diminish some of the negative effects of trauma during this crucial period in the life of both parents and infants.
